# Debate: Are cluster headache and migraine distinct headache disorders?

**DOI:** 10.1186/s10194-022-01504-x

**Published:** 2022-11-29

**Authors:** Mohammad Al-Mahdi Al-Karagholi, Kuan-Po Peng, Anja Sofie Petersen, Irene De Boer, Gisela M. Terwindt, Messoud Ashina

**Affiliations:** 1 Danish Headache Center, Department of Neurology, Rigshospitalet Glostrup, Faculty of Health and Medical Sciences, University of Copenhagen, Valdemar Hansen Vej 5, DK-2600 Glostrup, Denmark; 2 Department of Systems Neuroscience, University Medical Center Hamburg-Eppendorf, Hamburg, Germany; 3 Department of Neurology, Leiden University Medical Center, Leiden, Netherlands

**Keywords:** CGRP, Nitric oxide, PACAP, Trigeminovascular system, Cranial autonomic symptoms

## Abstract

Cluster headache and migraine are regarded as distinct primary headaches. While cluster headache and migraine differ in multiple aspects such as gender-related and headache specific features (e.g., attack duration and frequency), both show clinical similarities in trigger factors (e.g., alcohol) and treatment response (e.g., triptans). Here, we review the similarities and differences in anatomy and pathophysiology that underlie cluster headache and migraine, discuss whether cluster headache and migraine should indeed be considered as two distinct primary headaches, and propose recommendations for future studies.

**Graphical Abstract:**

Video recording of the debate held at the 1st International Conference on Advances in Migraine Sciences (ICAMS 2022, Copenhagen, Denmark) is available at https://www.youtube.com/watch?v=uUimmnDVTTE. 
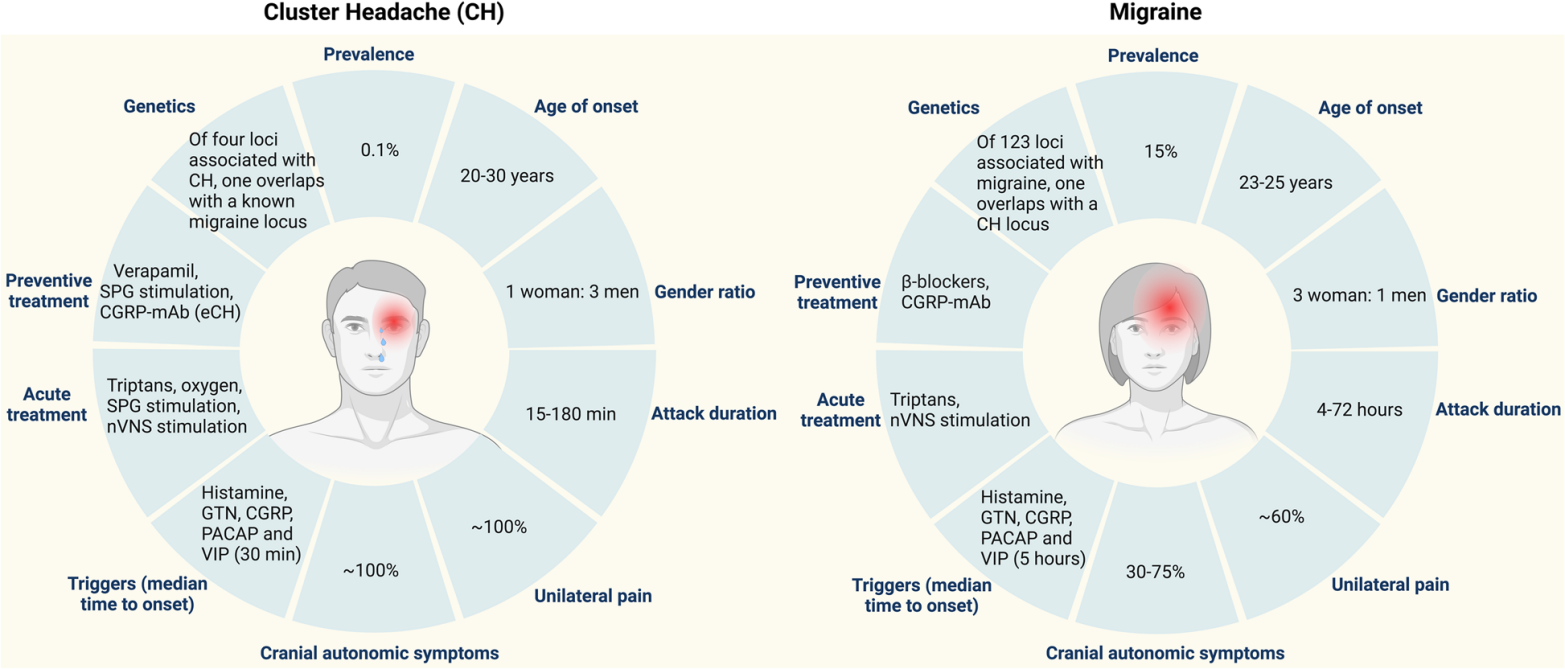

## Introduction

In the International Classification of Headache Disorders (ICHD-3), cluster headache (CH) and migraine are categorized as primary headaches [1]. CH and migraine affect 0.1% and 15% of the general population, respectively [2, 3]. CH is more common in men (men to women ratio ~ 4.3:1) [4, 5], while migraine primarily affects women (women to men ratio ~ 3:1). The prevalence of migraine in individuals diagnosed with CH does not differ from the general population [5]. Important clinical differences between cluster headache and migraine headache include duration and frequency of attacks. A CH attack lasts between 15 and 180 minutes, and multiple attacks per day may occur, whereas the duration of a migraine attack is between 4 and 72 hours, and recurrence is defined as a headache within 22 hours of initial successful treatment of a migraine attack (2-hour headache response) [6]. Furthermore, CH attacks are often side-locked, occurring on one side most of the times [7], while migraine headache localization changes or may be bilateral [8]. Interestingly, both share some non-headache related symptoms such as photophobia or cranial autonomic symptoms (CAS), although these may be more pronounced in one or the other [9]. Occasionally, some patients report an intermediate phenotype that includes specific features of both primary headaches or has comorbid CH and migraine [10]. In such patients, the attack duration, the presence of restlessness vs. pain aggravated by physical activities, and a family history of CH may provide diagnostic clues to distinguish between CH and migraine [11]. These similarities and differences between CH and migraine give rise to a debate about whether CH and migraine should be considered part of the clinical headache continuum or whether they are two distinct primary headaches.

### Phenotype

**Fig. 1:**
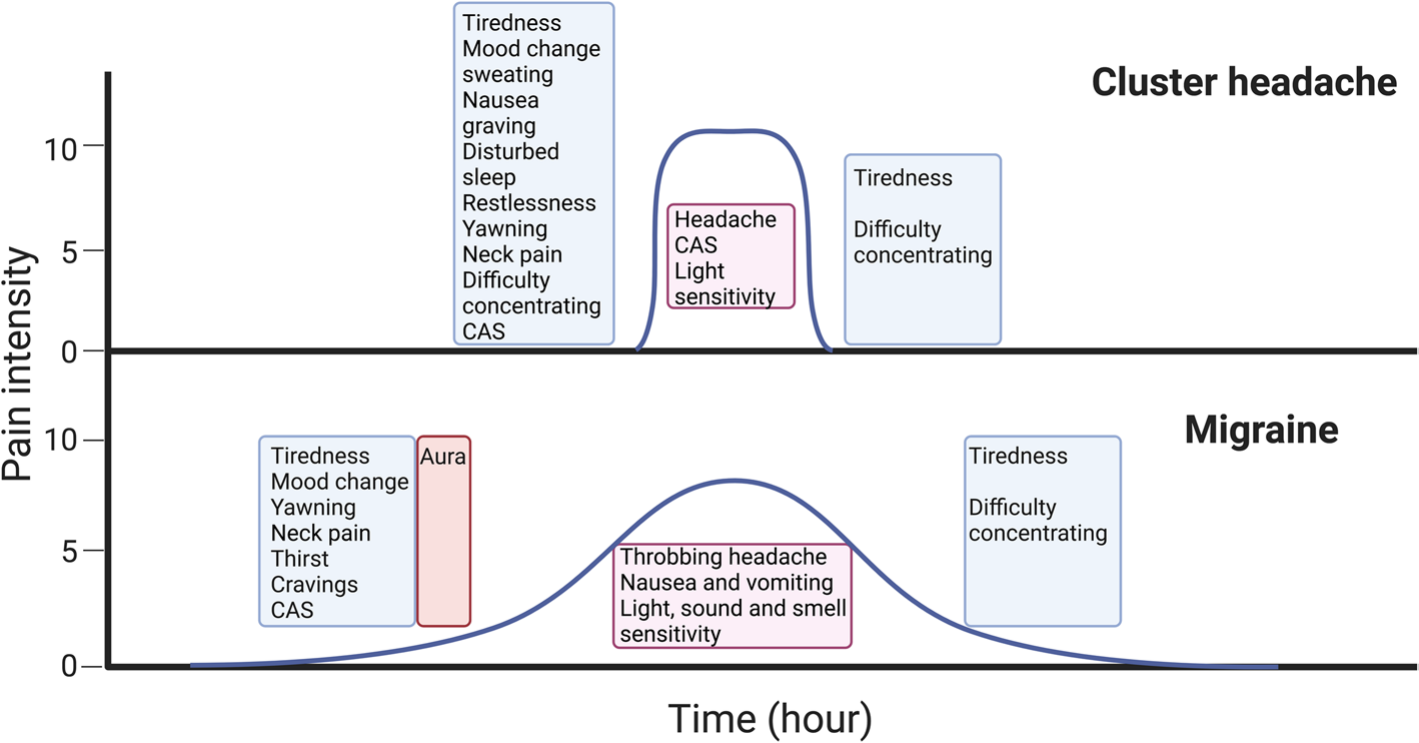
Clinical manifestation. Migraine is known for prodromal symptoms such as yawning, mood and cognitive changes, and neck pain which precede migraine headache for up to 2–3 days. Interestingly, Cluster headache (CH) patients also report prodromal symptoms which differ from those of migraine in their duration (up to one hour before CH attack). Eventually, migraine aura usually precedes the migraine headache, while the aura in CH patient is caused by a comorbid migraine with aura [12]

**Fig. 2:**
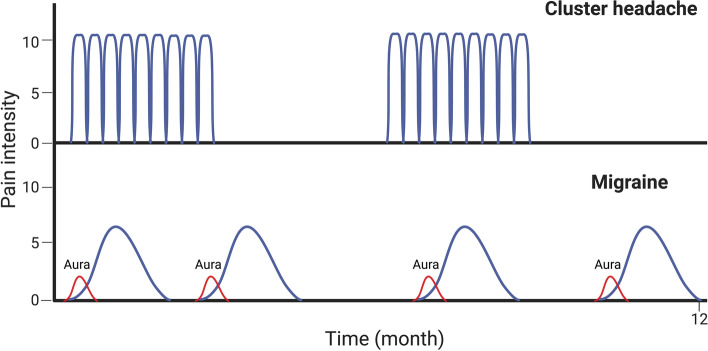
Chronobiologic rhythms. Cluster headache (CH) shows specific chronobiological features, mainly circadian (most frequently nocturnal) and circannual rhythms. In episodic CH, the attacks appear in a series of daily attacks lasting for weeks or months (cluster bout) followed by a complete remission for months or years. Migraine attacks rarely affect sleep and frequently occur during the day. Although migraine patients experience periodicity in attack frequency and severity [15], the periodicity of migraine attacks is not prominent compared to CH attacks

Clinical presentation of CH and migraine are shown in Tables 1 and 2. CH attacks are characterized by recurrent severe to very severe side-locked headaches associated with prominent ipsilateral CAS and/or agitation (Fig. 1). Attack frequency in CH ranges from one attack every other day to eight attacks a day [13, 14] with specific chronobiological features, mainly circadian (most frequently nocturnal) and circannual rhythms. In episodic CH, the attacks occur in a series of daily attacks lasting weeks or months (cluster bout) followed by a complete remission for months or years (Fig. 2) [14]. The age at onset of CH ranged from 10–68 years of age [16], with a peak between 20–30 years of age for both sexes (observed in ~40% of patients) [14]. Onset declines between 31–40 years of age (observed in 16% of patients) and between 41–50 years of age (observed in 10% of patients) [14].

**Table 1:** ICHD-3 Diagnostic criteria for cluster headache

**Cluster headache**	
**A.** At least five attacks fulfilling criteria B–D **B.** Severe or very severe unilateral orbital, supraorbital and/or temporal pain lasting 15–180 minutes (when untreated) **C.** Either or both of the following: 1. At least one of the following symptoms or signs, ipsilateral to the headache: a) Conjunctival injection and/or lacrimation b) Nasal congestion and/or rhinorrhoea c) Eyelid oedema d) Forehead and facial sweating e) Miosis and/or ptosis 2. A sense of restlessness or agitation **D.** Occurring with a frequency between one every other day and eight per day **E.** Not better accounted for by another ICHD-3 diagnosis.	
**Episodic cluster headache**	
**A.** Attacks fulfilling criteria for cluster headache and occurring in bouts (cluster periods) **B.** At least two cluster periods lasting from seven days to one year (when untreated) and separated by pain-free remission periods of ≥3 months.	
**Chronic cluster headache**	
**A.** Attacks fulfilling criteria for cluster headache and occurring in bouts (cluster periods) **B.** Occurring without a remission period, or with remissions lasting <3 months for at least one year.

**Table 2:** ICHD-3 Diagnostic Criteria for Migraine

**Migraine without aura**	
**A.** At least five attacks fulfilling criteria B–D **B.** Headache attacks lasting 4–72 hours (when untreated or unsuccessfully treated) **C.** Headache has at least two of the following four characteristics: 1. Unilateral location 2. Pulsating quality 3. Moderate or severe pain intensity 4. Aggravation by or causing avoidance of routine physical activity (e.g. walking or climbing stairs) **D.** During headache at least one of the following: 1. Nausea and/or vomiting 2. Photophobia and phonophobia **E.** Not better accounted for by another ICHD-3 diagnosis.	
**Migraine with aura**	
**A.** At least two attacks fulfilling criteria B and C **B.** One or more of the following fully reversible aura symptoms: 1. Visual 2. Sensory 3. Speech and/or language 4. Motor 5. Brainstem 6. Retinal **C.** At least three of the following six characteristics: 1. At least one aura symptom spreads gradually over ≥5 minutes 2. Two or more aura symptoms occur in succession 3. Each individual aura symptom lasts 5–60 minutes 4. At least one aura symptom is unilateral 5. At least one aura symptom is positive 6. The aura is accompanied, or followed within 60 minutes, by headache **D.** Not better accounted for by another ICHD-3 diagnosis.	
**Chronic migraine**	
**A.** Headache (migraine-like or tension-type-like) on ≥15 days/month for >3 months, and fulfilling criteria B and C **B.** Occurring in a patient who has had at least five attacks fulfilling criteria B–D for migraine with- out aura and/or criteria B and C for migraine with aura **C.** On ≥8 days/month for >3 months, fulfilling any of the following: 1. Criteria C and D for migraine without aura 2. Criteria B and C for migraine with aura 3. Believed by the patient to be migraine at onset and relieved by a triptan or ergot derivative **D.** Not better accounted for by another ICHD-3 diagnosis.

**Fig. 3:**
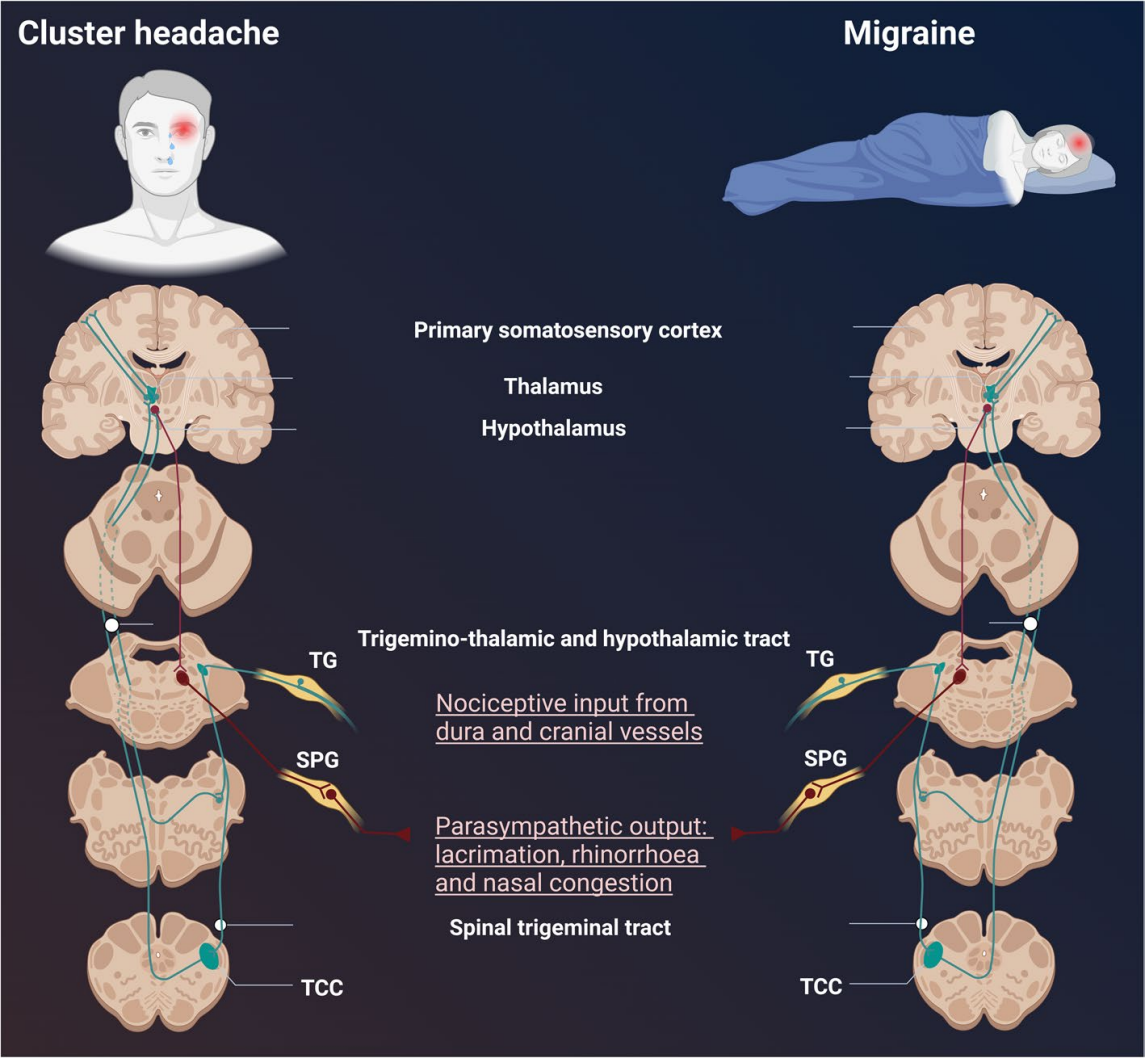
Trigeminovascular system (TVS). Generation of Cluster headache (CH) involves the trigeminocervical complex (TCC), the parasympathetic nerve fibers (trigeminal autonomic reflex (TAR)), and the hypothalamus [21–29]. Peripheral fibers of neurons in the trigeminal ganglions (TG) transmit nociceptive information from dura mater and cranial vessels to the TCC in the brainstem. Fibers from the TCC project to thalamic neurons (via the trigemino-thalamic tract) and to hypothalamic neurons (via the trigemino-hypothalamic tract). Neurons within the TCC are connected to parasympathetic neurons in the superior salivatory nucleus, and the activation of the parasympathetic system by the trigeminal neurons comprises the TAR. The parasympathetic fibers from the superior salivatory nucleus pass through the facial nerve and the sphenopalatine ganglion (SPG) on the way to the periphery. Release of neuropeptides upon activation of the parasympathetic system causes autonomic symptoms such as cephalic vasodilation, conjunctival injection, lacrimation and rhinorrhea. Clinical experience indicates involvement of TAR in CH more than migraine. This notion is further strengthened by the finding that low frequency SPG stimulation induced CH attacks with autonomic features, which could subsequently be treated by high frequency SPG stimulation [30]. Low frequency stimulation of the SPG did not induce migraine attacks or autonomic symptoms in migraine patients. These data suggest that increased parasympathetic outflow by (SPG) neurostimulator does not initiate migraine attacks [31]. However, a recent study demonstrated that low frequency SPG stimulation induced autonomic features but no CH attacks [32]. The clinical manifestation of CH attacks, including circadian rhythm dependence, relapsing– remitting presentations and ipsilateral cranial autonomic symptoms indicate hypothalamic involvement. While the anterior hypothalamus might contribute to the circadian rhythm of CH attacks, the lateral and posterior part might generate the restlessness experienced by CH patients during the attack. Neuroimaging investigations report a role of the hypothalamus during the prodrome symptoms and dorsolateral pons during the ictal phase of attacks in individuals with migraine patients. CH and migraine seem to share anatomical structure with distinct biology

Migraine attacks are characterized by recurrent unilateral moderate to severe pulsating headaches, aggravated by routine physical activity. Strictly unilateral (side-locked) headache are reported in approximately 26% of migraine patients [17], and up to 40% of the individuals with migraine reported bilateral headache [18]. Migraine is a life span disease with an age-dependent change. The prevalence of migraine increases with age and peaks at 35–39 years of age, followed by a decline [19]. These changes may include transformation from episodic to chronic migraine or even a disappearance of some or all migraine symptoms [20]. Although, seasonal variation of migraine attacks is less prominent and migraine attacks are more equally distributed compared to CH attacks (Fig. 2), some patients experience periodicity and report increased frequency of attacks at certain times of the year [15]. Migraine attacks rarely affect sleep and frequently occur during the day (Figs. 2 and 3). CH and migraine may coexist in the same patient. Cross-sectional cohort studies reported comorbid migraine in 10–16.7% of patients with CH [33–35]. Notably, the proportion is similar to the prevalence of migraine in the general population [3]. Comorbid CH in migraine cohorts has yet to be investigated. This partially reflects the relatively low prevalence of CH in the general population [2]. Whether the comorbidity suggests a shared disease mechanism or a co-occurrence by chance requires further investigation, especially in longitudinal studies.

Ipsilateral CAS with an average of four symptoms [14] have been reported in >90% of patients with CH (Table 3) [48]. Migraine patients may also experience CAS, but studies reported a wide range of prevalence 30–75% [49–51]. The average of CAS during migraine attack is 2 symptoms/attacks [45], which is equivalent to half of what a CH patient experiences, even though it has never been investigated head-to-head. Photophobia defined as enhanced sensitivity to light is one of the most typical associated symptoms of migraine reported in ≥80% of migraine patients [42], Of note, 80% of patients with CH report photophobia during their attacks (Table 3) [43, 52]. Visual allodynia defined as enhanced sensitivity to light and patterns was recently investigated in CH. Interestingly, CH patients mostly report unilateral visual allodynia that is ipsilateral to the side of the ictal pain [53, 54]. Cutaneous allodynia is a common feature accompanying migraine attacks [55] and is considered a clinical marker of central sensitization [38], and a risk factor for migraine chronification [38] but not associated with chronic CH [41]. Interestingly, 36% of patients with CH report allodynia during attacks [41]. Female gender, young age at onset, lifetime depression, comorbid migraine, and recent attacks were independent risk factors for allodynia. The high prevalence of cutaneous allodynia with similar risk factors for allodynia as found for migraine suggests that central sensitization, as with migraine, also occurs in CH [41]. However, it remains to be seen whether the presence of allodynia in CH has a predictive value for treatment response. An important clinical difference that distinguishes CH from migraine is the restlessness, which causes patients to wander during attacks [46, 47]. While light physical activity exacerbates migraine headache, and migraine patients usually lie down during attacks (Table 3) [1].

**Table 3:** Clinical presentation. Comparison of clinical presentation between cluster headache and migraine

	Migraine	Cluster headache
Unilateral pain	60%	100%
Intensity	Moderate to severe	Severe – very severe
Duration	4–72 hours	15–180 minutes
Circadian rhythm	Less prominent	Prominent
Presence of prodromes	83.3% [36]	72% [37]
Ictal allodynia	40–70% [38–40]	36% [41]
Photophobia (ictal)	80% [42]	91% [43]
Phonophobia (ictal)	98% [44]	89% [43]
Cranial autonomic symptom Restlessness	74% [45] Physical activity usually worsens headache	Nearly 100% 70% [46]-88% [47]

Migraine patients may experience prodromal symptoms such as yawning, changes in mood and difficulty concentrating, as well as neck pain which precede migraine headache by up to 2–3 days (Fig. 1) [56]. In contrast, similar prodromal symptoms in CH precede attacks by up to one hour (Fig. 1) [36, 57]. In the case of aura, migraine aura usually precedes the migraine headache, while the aura in CH patient is often caused by a comorbid migraine with aura (Fig. 2) [12]. Thus, the clinical manifestations of both primary headaches overlap to some extent; however, the striking circannual and circadian periodicity, duration of attacks and some associated symptoms are clearly different (Fig. 2).

### Disease Mechanisms

#### Genetics

The risk for first-degree relatives of CH patients to develop CH is estimated to be 5–18 times higher than that of the general population [58], while the risk for first-degree relatives of migraine patients to develop migraine is estimated to be 1.9- (migraine without aura) and 3.8-fold increased (migraine with aura), compared to the risk in the general population [59]. However, although we cannot exclude that some patients might inherit CH in a mendelian fashion, multifactorial inheritance, as is almost always also the case in migraine, seems likely [60, 61]. For a long time, while we increasingly understood the genetic architecture of migraine, the genetic basis of CH remained a mystery. Whether there is a genetic overlap between them remained a conundrum.

The latest genome-wide association study (GWAS) of migraine found 123 loci, of which 86 were previously unknown [62]. Here, 102,084 migraine cases and 771,257 controls were analyzed. Two recent GWAS studies independently identified the first four replicating genomic loci associated with CH (even though less than 1500 CH patients were included per study) [63, 64]. Interestingly, one of the associated loci, located on chromosome 6, which covers both *FHL5* and *UFL1,* overlaps with a previous known migraine locus. Moreover, the association had the same effect direction for both CH and migraine. Notedly, the effect sizes were higher for CH (OR≈1.30) than for migraine (OR≈1.09) for this locus [63, 64]. The larger effect size for CH makes it unlikely that misclassification and comorbid migraine causes this identified association and suggests that this locus has a greater effect on risk of developing CH than migraine. The effect size might also be influenced by the CH populations, that were very homogenous and had validated diagnosis according to the ICHD-criteria. To date, no other migraine loci have been identified to associate with CH (36 other loci from the migraine 2016 meta-analyses were tested) [63, 64]. So, while CH and migraine might partly share their genetic architecture, they probably also have distinct genetic components. This may suggest both partly shared and partly distinct involved biological mechanisms.

#### Pathophysiology

The trigeminovascular system (TVS) is the anatomical and physiological substrate of CH [7] and migraine [65] (Fig. 3). Activation of the TVS is associated with release of various vasoactive neuropeptides, including calcitonin gene-related peptide (CGRP), pituitary adenylate cyclase-activating polypeptide-38 (PACAP38) and vasoactive intestinal polypeptide (VIP). To explore signaling pathways within the TVS, several pharmacological compounds were used to induce CH attacks and migraine attacks including histamine, glyceryl trinitrate (GTN), CGRP, PACAP38 and VIP [66–68].

Pharmacological triggers for migraine attacks are effective triggers for CH attacks (Table 4). In randomized placebo-controlled clinical trials (RCT), GTN and CGRP induced CH attacks [69, 70]. One RCT investigated PACAP38 and VIP head-to-head in the same CH patients [71]. Interestingly, CH attacks are triggered faster (~30 min, range 10–90 min) compared to migraine attacks (~5 hours, 2–11 hours) (Table 4). Additionally, the induction rate of CH attack is highly dependent on the disease phase: episodic in-bout and chronic versus out-of-bout (remission). Intravenous infusion of CGRP induced CH in episodic and chronic CH patients but patients in remission phase reported no attacks. Interestingly, CH attacks in chronic patients are less likely to be triggered, while the potency of CGRP as a migraine inductor is increased in chronic migraine patients with ongoing headache [72]. These observations suggest that CH and migraine share anatomical structures and pathophysiological mechanisms but differ in signaling cascades leading to attack initiation. Notably, participants diagnosed with other types of headaches including persistent post-traumatic headache are also hypersensitive to CGRP [73], indicating that CGRP has an integral role in the pathogenesis of headache in general and not specific for CH and migraine.

**Table 4:**
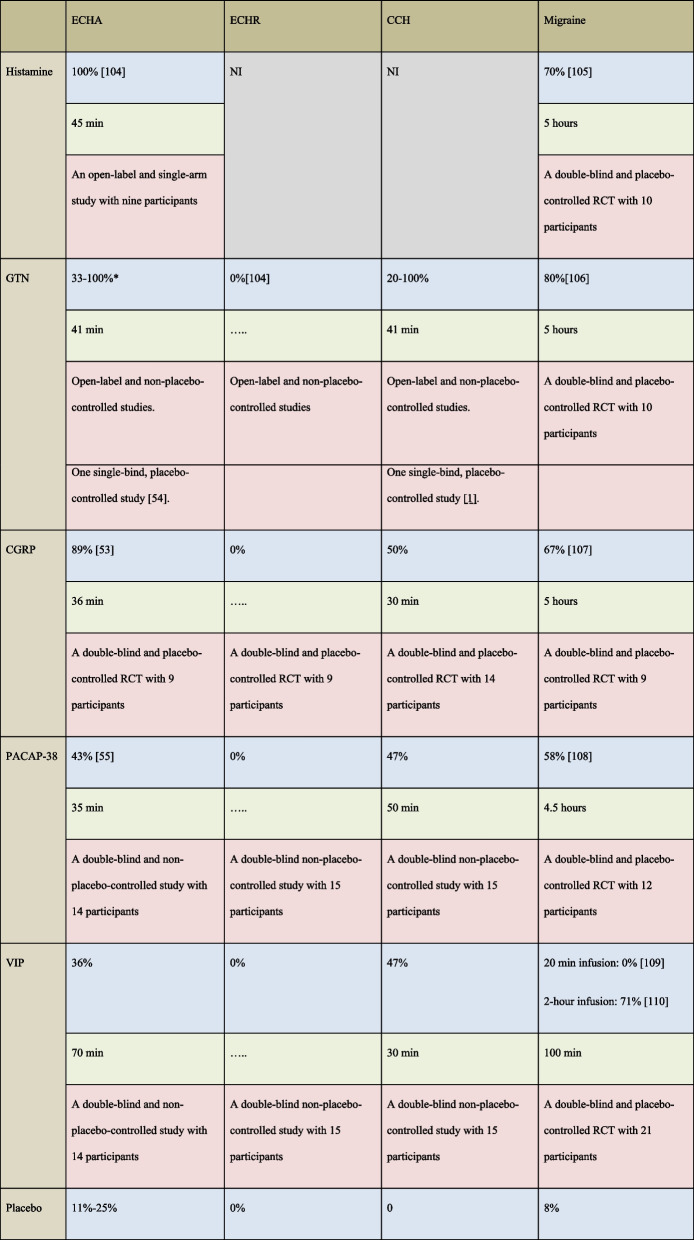
Provocation studies. Cluster headache and migraine have several pharmacological triggers in common but different methodological approach have been applied in attack induction

Blue indicates induction rate, green indicates median time to onset, and red indicates study design. *RCT* Randomized clinical trial, *ECHA* Episodic cluster headache in active phase, *ECHR* Episodic cluster headache in remission phase, *CCH* Chronic cluster headache, and *NI* Not Investigated

Gender-related etiology of CH and migraine suggests that sex hormones are affected in both disorders. It is reported that male patients with CH exhibited decreased levels of testosterone [74], and male patients with migraine exhibited increased levels of estradiol and showed a clinical evidence of relative androgen deficiency compared to controls [75]. Yet, the influence of sex hormone is complex, and more insight is needed to make conclusive comments on the similarities and differences.

#### Prodromal symptoms and imaging

Longitudinal human studies showed a significant hypothalamic activation up to 48 hours before migraine headache [76, 77]. Although no prodromal symptoms were recorded, these studies concluded that hypothalamus is linked to prodromal symptoms preceding the ictal phase of migraine attacks [76, 77]. Imaging studies showed that other brain regions were activated, such as the midbrain tegmental area and periaqueductal grey [78]. To date, no functional imaging studies have investigated CH patients during prodromal symptoms. The hypothalamus is activated during the ictal period of CH attacks [79]. A recent fMRI study revealed an activation of the posterior hypothalamus by trigeminonociceptive stimuli in CH patients during remission, suggesting an important role of the hypothalamus, even outside the headache attacks [80]. Interestingly, the anterior hypothalamus is activated in patients with chronic migraine [81] and chronic CH [82]. Given that the hypothalamus modulates chronological rhythm [83] and its specific subnuclei may explain prodromal symptoms [83], it would be plausible to suggest the hypothalamus may also play an important role in the genesis of migraine and CH attacks.

#### Treatment

Management of CH and migraine involve acute and preventive treatments. Triptans are serotonin agonists which target 5-HT_1B_ and 5HT_1D_ receptors [84]. Since the pharmacodynamics of triptans are rather specific and do not involve the antinociceptive activity against noxious stimuli, triptans are ineffective in non-cephalic pain conditions [85]. RCTs showed that triptans are effective as acute therapies for migraine [86] and CH attacks [87] (Table 5). Oxygen therapy (inhalation of 100% oxygen through a face mask with a flow of 12–15 L/min) is widely used to relieve acute pain during CH attacks [21]. The exact underlying mechanism for this effect is uncertain, and several explanations have been proposed, including inhibition of the trigeminoautonomic reflex (TAR), modulation of neurotransmitters, and cerebral vasoconstriction [88–90]**.** To date, no RCT has assessed the efficacy of oxygen therapy in migraine patients.

**Table 5:** Treatment. Comparison of treatment responses between cluster headache and migraine

	Migraine	Episodic cluster headache
Triptan	+++	+++
CGRP-mAb	+++	++/−
Oxygen	++	+++
Steroid	++	++
Topiramate	+++	+
Melatonin	+++	++
nVNS	++	++
SPG modulation	++/− (chronic migraine)^a^	++ (chronic cluster headache)^b^

+++ efficacy proved in ≥2 randomized placebo-controlled studies

++ efficacy proved in 1 randomized placebo-controlled study

+ open label studies

- negative randomized placebo-controlled study

*nVNS* Non-invasive vagus nerve stimulation, *SPG* Sphenopalatine ganglion

^a^SPG block

^b^SPG stimulation

The first-line CH preventive treatment verapamil [91] has only slight efficacy in migraine prevention [92]. Candesartan, an angiotensin II receptor antagonist, showed effectiveness in migraine prevention [93] but failed to prevent CH [94]. Inhibition of the parasympathetic outflow by sphenopalatine ganglion (SPG) stimulation showed dual beneficial effects, acute pain relief and attack prevention in CH [22]. In contrast, migraine patients did not report any meaningful response after SPG stimulation [95]. Non-invasive vagus nerve stimulation (nVNS) showed significant efficacy in aborting migraine attacks [96] and attacks in episodic CH, but not attacks in chronic CH [97].

Anti-CGRP monoclonal antibodies (CGRP-mAb) including galcanezumab and fremanezumab are novel mechanism-based therapies developed for migraine prevention [98]. Four RCTs assessed the safety and efficacy of CGRP-mAb to prevent CH. In episodic CH, galcanezumab reduced CH attacks by 3.5 per week (95% CI: 0.2–6.7, *p* = 0.04) [99]. In chronic CH, galcanezumab did not meet its primary and key secondary endpoints [100]. Clinical trials with fremanezumab (NCT02945046 with episodic and chronic CH participants; and NCT02964338 with chronic CH participants) were discontinued due to the negative results of the mid-term futility analysis. These conflicting findings highlight the irregularity and unpredictability of cluster periods across participants and the spontaneous remission as part of the natural history of episodic CH [101]. Interestingly, treatment efficacy differs greatly between episodic and chronic CH patients. Patients with chronic CH were less likely to respond to intranasal zolmitriptan [102] or oxygen therapy [103]. Verapamil is almost 50% less likely to be effective in patients with chronic CH compared to those with episodic CH [104]. Additionally, none of the new treatment options, such as CGRP-mAb or nVNS, have been shown to be effective in chronic CH [97, 100], despite efficacy in patients with episodic CH [97, 99]. One explanation for these observations is that chronic CH patients have a low threshold and are thus more susceptible to recurrent attacks. Another possible explanation would be a different neurobiology. For example, chronic CH patients, in addition to a circadian rhythm, have an additional ultradian rhythm – a period ≤24 h and averaged 4.8 h in one study [105], and serum CGRP levels were lower in chronic patients than episodic patients [106]. Taken together, CH and migraine share clinical efficacy to treatment options (Table 5) with a specific mechanism of action.

#### Lessons Learned and Future Directions

CH and migraine appear to have a strong genetic component. The latest CH GWASs indicated that they share at least one genetic locus. Increasing sample size (mainly for the CH cohorts currently available for analyses) and meta-analyses of the genetic data available will further elucidate shared and distinct genetic components of the disorders. Despite the abundance of shared clinical features between CH and migraine, none of the headache features are specific to any headache diagnosis. For example, photophobia is not restricted to CH or migraine [107]. Patients with secondary headaches including post-traumatic headache and headache attributed to intracranial infection (e.g. meningitis) may report photophobia and other clinical manifestation that mimic primary headaches [108, 109]. Thus, none of the clinical features are diagnosis-specific and possibly simply reflects the activation of the trigeminal pain pathway. The presence or absence of certain associated symptoms may reflect the degree of activation: e.g., CAS might only accompany severe headaches. The most striking characteristic of CH is the short attack duration. Regardless of the severity and intensity of the attack, the attack stops spontaneously within 180 minutes. The mechanism of how cluster and migraine attacks stop spontaneously remains unknown. In discussing the structures and molecules involved in CH and migraine, numerous questions remained to be answered: 1) molecular pathways responsible for genesis of attacks; 2) factors modulating susceptibility to attacks; 3) the precise mechanisms and order of events behind the initiation of attacks; 4) molecular pathways underlying attack termination. Pharmacological provocation studies in both CH and migraine provided valuable information on molecular signaling pathways. Recent studies that targeted the downstream signaling pathway in the vascular smooth muscles are intriguing: the opening of ATP-sensitive potassium (K_ATP_) channels [110] or high-conductance (big) calcium-activated potassium channels (BK_Ca_) channels [111] served as highly effective migraine attack triggers (95–100%). Clinical trials in patients with CH are still ongoing (NCT05093582), and such studies are critical in deciphering the genesis of CH attacks. Functional imaging studies are known to be influenced by the study site, study design, and even analytical methods [112]. Studies using resting-state fMRI are highly depend on participants’ alertness and are rarely reproducible [113]. To reduce inter-study and even inter-session differences, headache-to-headache comparison between CH and migraine will be necessary, and these studies are still lacking. Furthermore, functional imaging studies investigating patients with CH are difficult to conduct because patients usually have restlessness during their attacks.

Although patients with CH and migraine share several specific treatment options, the mechanism or the site of action remains largely uncertain. Another critical question is whether drug response should be used to assist diagnose and classify headache disorders? Response to the drug has only been adopted as a diagnostic criterion in paroxysmal hemicrania and hemicrania continua. In addition, for any given medication, there are always clinical responders vs. clinical non-responders. The diverse response to a specific medication suggests that the clinical cohort, e.g., migraine patients, can still be divided into those with distinct molecular mechanisms (and hence different response to specific treatment).

## Conclusions

CH and migraine share some clinical features, including non-headache (pre) ictal features such as prodromal features, (inter) ictal visual hypersensitivity, ictal allodynia and cranial autonomic features. Demographics, genetics and chronological patterns suggest partly overlap, but also important differences in pathophysiological mechanisms. Common pharmacological triggers suggest shared anatomical and pathophysiological substrate, but as CH attacks are triggered faster compared to migraine attacks, signaling cascades leading to attack initiation might differ. More studies are needed to improve understanding of the disease mechanism of CH and migraine. It is also crucial to discover potential biomarkers, with which we may better categorize the disease entity and help identify the susceptible group for specific treatment options.
